# The Impacts of Spatiotemporal Landscape Changes on Water Quality in Shenzhen, China

**DOI:** 10.3390/ijerph15051038

**Published:** 2018-05-22

**Authors:** Zhenhuan Liu, Haiyan Yang

**Affiliations:** 1 Guangdong Provincial Key Laboratory of Urbanization and Geo-simulation, School of Geography and Planning, Sun Yat-sen University, Guangzhou 510275, China; liuzhh39@mail.sysu.edu.cn; 2 College of Water Conservancy and Civil Engineering, South China Agricultural University, Guangzhou 510642, China

**Keywords:** landscape changes, water quality, rapid urbanization, panel regression analysis, Shenzhen

## Abstract

The urban landscape in China has changed rapidly over the past four decades, which has led to various environmental consequences, such as water quality degradation at the regional scale. To improve water restoration strategies and policies, this study assessed the relationship between water quality and landscape change in Shenzhen, China, using panel regression analysis. The results show that decreases in natural and semi-natural landscape compositions have had significant negative effects on water quality. Landscape composition and configuration changes accounted for 39–58% of the variation in regional water quality degradation. Additionally, landscape fragmentation indices, such as patch density (PD) and the number of patches (NP), are important indicators of the drivers of water quality degradation. PD accounted for 2.03–5.44% of the variability in water quality, while NP accounted for −1.63% to −4.98% of the variability. These results indicate that reducing landscape fragmentation and enhancing natural landscape composition at the watershed scale are vital to improving regional water quality. The study findings suggest that urban landscape optimization is a promising strategy for mitigating urban water quality degradation, and the results can be used in policy making for the sustainable development of the hydrological environment in rapidly urbanizing areas.

## 1. Introduction

Water quality degradation is a key issue for global environmental change in urban areas and a serious problem in many rapidly urbanizing catchments in developing countries [1,2] where wastewater is continuously discharged to stream systems due to highly centralized socioeconomic development and anthropogenic processes [3]. Urbanization alters landscape patterns that, in turn, control the various biogeochemical and physical processes of watersheds [4]. Thus, it is important for researchers of urban sustainability and river ecosystem restoration to understand the relationship between landscape change and water quality at the watershed scale. Landscape governance in urban areas may provide a scientific foundation for addressing the effects of urbanization on water pollution [5].

Several recent studies have addressed the interactions between water quality degradation and landscape changes [6], with a focus on the selection of suitable landscape metrics, water quality parameters, scales, and statistical methods as they relate to water quality and the landscape [7]. Simple statistical analysis at the watershed scale considers the effect of the spatial configuration of the landscape as an important factor in understanding the hydrological processes related to land use and water quality in adjacent aquatic systems [8,9]. However, such analyses are limited by the need to quantify the changes in the landscape and water quality. As simple statistical metrics, landscape indicators are commonly used to quantify the spatial relationships between water quality variables [10,11]. Although these studies have become more common in recent years, many associated questions remain unanswered. For example, the quantitative contributions of landscape changes to variations in urban stream water remain unknown. Therefore, it is necessary to combine spatial changes and temporal changes through statistical analyses to determine the relationship between water quality and the landscape on a regional scale.

Most previous studies have emphasized the relationship between landscape patterns and water quality while neglecting the synergistic effects of landscape changes on water quality. When researchers conduct related analyses, they often neglect temporal landscape change information and apply statistical spatial information using various techniques, such as Pearson’s correlation analysis [6], spatial autocorrelation [12], geographically weighted regression [13], redundancy analysis [14] or de-trended correspondence analysis [15]. Other studies have focused on the effects of landscape patterns at different scales on water quality using linear regression [10,11], generalized linear mixed regression [16], logistic regression [17], nonlinear regression [18] or stepwise regression analysis [19]; however, these studies did not consider changes in landscape patterns. The relationship between landscape change and water quality degradation is not clear, especially during rapid urbanization [20] and most previous studies are complicated by static landscape patterns [21]. It is difficult to fully explain the contributions of landscape changes to water quality using a correlation relationship. Landscape changes include information on landscape composition and configuration and the interactions that affect water quality. Panel regression analysis can be used to effectively assess the synergetic relationship between spatiotemporal landscape change and water quality. In addition, this approach can yield the correlation between landscape change and water quality, as well as the contributions of landscape change to water quality [22]. This approach has considerable potential in spatiotemporal data analysis and is a very effective method of quantifying coupled influences.

Seasonal and annual variability in watersheds are two important timescales of water quality changes [23]. Many studies of the relationship between landscape patterns and water quality have focused only on seasonal variations or short-term impacts. Hydrological and biogeochemical processes, such as surface runoff and the nitrogen and phosphorus cycles are important mechanisms that can explain these seasonal or short-term changes [12]. However, landscape changes mainly influence the annual variations in the water quality of urban stream systems. If watershed management in a region is designed to balance development with water quality, it is necessary to quantify the extent to which water quality degradation is caused by the spatial and temporal variability of landscape changes, as these changes have different rates, and the environmental consequences differ in different watersheds. The objectives of this study were as follows: (1) to quantify the spatial and temporal variations in water quality in Shenzhen based on multi-statistical analysis and identify the spatial and temporal degradation processes of different watersheds in Shenzhen; (2) to assess the relationships between landscape change and water quality degradation from 1990 to 2010 by considering water quality degradation stages and spatial characteristics using panel regression analysis; and (3) to address the contributions of landscape changes to water quality changes during rapid urbanization.

## 2. Materials and Methods

### 2.1. Site Description

**Figure 1:**
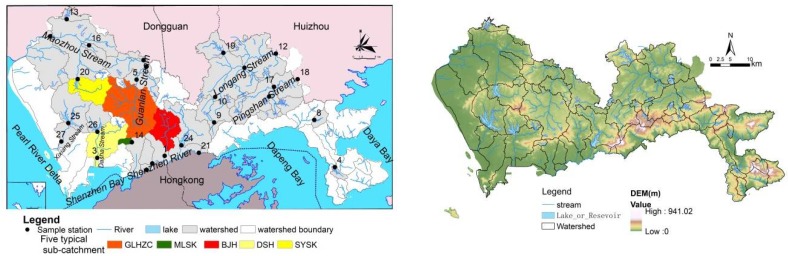
Sample stations and their corresponding watersheds and streams in Shenzhen, (**left**) Sample stations and the sub-catchment; (**right**) Digital elevation model (DEM).

Shenzhen, the first Special Economic Zone, is a new city that formed after the Chinese reform and opening policies were issued. More than 45% of the natural landscape in Shenzhen has been converted to an urban landscape over the past 40 years due to rapid urbanization [24]. The consequences of rapid urbanization in Shenzhen have been extensively researched in environmental and ecological resource studies, which have focused on landscape urbanization [25], impervious surface area expansion [20], habitat fragmentation [26], urban heat island effects, vegetation degradation and ecosystem health deterioration [27]. One of the largest environmental pollution problems is the deterioration of water quality in small catchments [18]. Shenzhen includes approximately 310 streams. Sixty-nine of these streams cover a watershed area of over 10 km^2^, including the Shenzhen River, Maozhou Stream, Longgang Stream, Guanlan Stream, and Pingshan Stream. In this study, 27 subwatersheds were selected in accordance with the sample locations. The government constructed many reservoirs and floodgates during the urbanization process to conserve water resources, and most of the selected sample sites are independent because they are not hydrologically connected (Figure 1). All of the sub-watersheds were selected within the Shenzhen area, and they did not overlap with the boundary of the overall watershed to minimize the sub-watershed differences in geological, climatic, geographic, and hydrological conditions [18].

### 2.2. Data Sources 

Water quality data were collected from over 51 sampling sites by the Environmental Protection Bureau of Shenzhen Municipality. However, only 27 sampling sites and corresponding sub-watersheds were used in this study because sub-watersheds were separated as independent hydrological systems. Overall, six water quality indicators were selected to assess the different chemical characteristics of streams: the chemical oxygen demand (COD_Mn_), 5-day biochemical oxygen demand (BOD_5_), ammonia nitrogen (NH_3_-N), total phosphorus (TP), volatile phenol (VP), and oils (Oils). All the above indicators are reported in units of mg/l. Sampling was conducted under low-flow, normal-flow, and high-flow conditions. Samples were collected twice during each flow period and six times per year (12 times per year in the reservoir watersheds). All sample data sets from 1990 to 2010 were obtained from the 1991–2012 Environmental Quality Bulletins by the Environmental Protection Bureau of Shenzhen Municipality.

**Figure 2:**
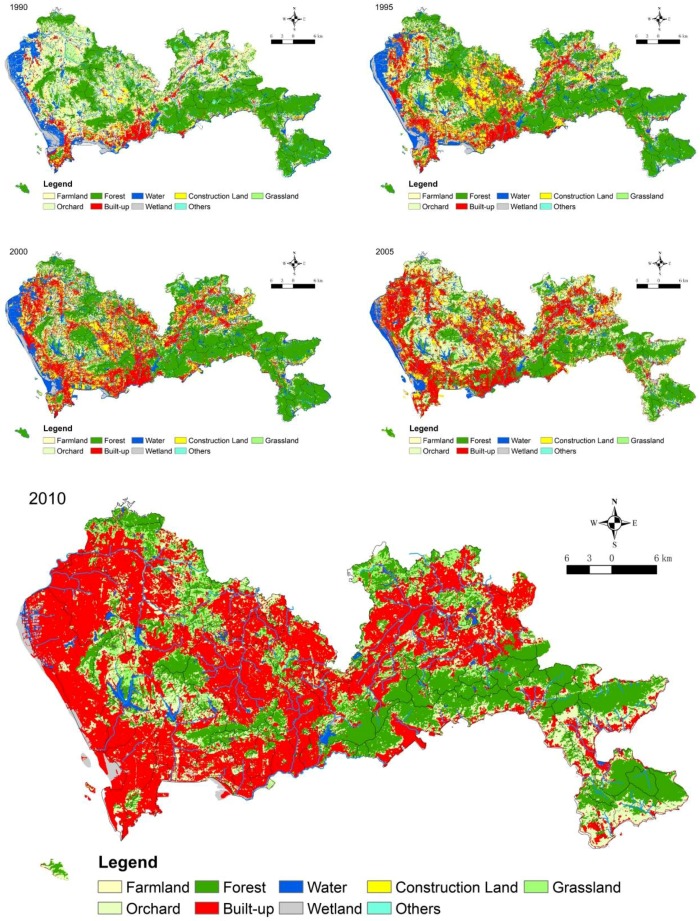
Landscape distributions in Shenzhen during 1990–2010.

The catchment landscape data were derived using Landsat TM/ETM+ images from 1990 to 2010 (from October to February) using maximum likelihood supervised classification algorithms, and the accuracy of the landscape data classification has been published in other studies [28]. The data were classified into nine landscape types: farmland (FL), orchard landscape (OL), forest landscape (FL), built-up landscape (BL), water landscape (WL), grass landscape (GL), wetland landscape (WetL) construction landscape (CL), and undeveloped landscape (UNDL). CL is a temporary landscape in urban areas that transitions to BL. Additionally, rapid urbanization often creates large areas of temporary construction land (Figure 2).

### 2.3. Multivariate Statistical Analysis

Cluster analysis (CA) using Ward’s method and the squared Euclidean distance were employed to describe the spatial and temporal patterns of water quality [29]. Based on water quality data and monitoring site codes, water quality can be grouped based on the spatial scale or temporal stage. Using a spatial scale, water quality sites can be clustered into similar urban development stages. Temporally, water quality time series can be divided into different development stages. In this study, boxplots were used to explain the variations in the water quality distribution.

### 2.4. Landscape Pattern Analysis

Ten metrics were chosen to quantify changes in the catchment landscape pattern at the landscape level based on the patch size, shape, and structure and the landscape diversity. These metrics included the number of patches (NP), cohesion (Cohesion), Shannon’s diversity index (SHDI), patch density (PD), landscape shape index (LSI), edge density (ED), interspersion and juxtaposition index (IJI), percentage of the cultivated landscape combined with the agricultural and orchard landscapes (CultiP), the percentage of FL (ForestP), and the percentage of UL combined with built-up and developing landscapes (UrbanP). These metrics reflect not only land use and land cover but also the spatial configuration and composition of the landscape at the catchment scale. These metrics have also been considered relative to water quality in previous studies [4,8,10,11]. All of the metrics were calculated using the FRAGSTATS 4.2 software (University of Massachusetts Amherst, Amherst, MA, USA) [30].

### 2.5. Panel Regression Analysis

Panel regression analysis was employed to examine the relationships between landscape patterns and water quality in urban catchments. Panel data analysis can be used to assess a variety of regression models and observation times, and it reduces the risk of collinearity between variables. Panel data analysis can incorporate observational or cross-sectional data, and the data can be temporally investigated. In addition, variable intercept panel data models can be advantageous for interpreting the specific impacts of spatial heterogeneity. In this study, a fixed-effect panel data model was used for data assessment based on the following equation:

(1) $$\log\left(y_{i,t}\right)=\sum_{j}\alpha_{j}\log\left({\mathrm{LCP}}_{i,j,t}\right)+\sum_{j}\beta_{j}\log\left({\mathrm{LCF}}_{i,j,t}\right)+\mu_{i}+\varepsilon_{i,t}$$

where $y_{i,t}$ is the dependent variable with measurement unit i (i = 1, 2, …, N) at time t (t = 1, 2, …, T); ${\mathrm{LCP}}_{i,j,t}\;$ are vectors of observations for m independent variables of landscape composition ([N × T] × m); $\;{\mathrm{LCF}}_{i,j,t}\;$ are vectors of observations for independent variables of landscape configuration; $\alpha_{j}$ and $\beta_{j}$ are the matching vectors of unknown model parameters; $\varepsilon_{i,t}$ is an independently and identically distributed error term with a zero mean and variance of σ^2^; and $\;\mu_{i}$ denotes a specific effect. In the case of water quality modeling, some space-specific variables that affect 5-year water quality degradation are omitted, such as socioeconomic factors. The random effects can be best interpreted based on the overall characteristics of the model results; therefore, a random effects model was employed in this study. All the variables were transformed into logarithmic form to reduce multicollinearity, eliminate the influence of the dimension, and investigate the rate of change and flexibility of each variable. The estimation results of a Hansman test indicated that the fixed-effects model is appropriate for assessing the interactions between variables (*p* > χ^2^ = 0.000).

## 3. Results

### 3.1. Spatial and Temporal Variations in Water Quality

The water quality statistics indicate that the mean values of the variables notably exceed the standard water quality level III in all instances and level V in some instances (Table 1). Furthermore, BOD_5_, NH_3_-N, and TP are the three main pollution indicators.

**Figure 3:**
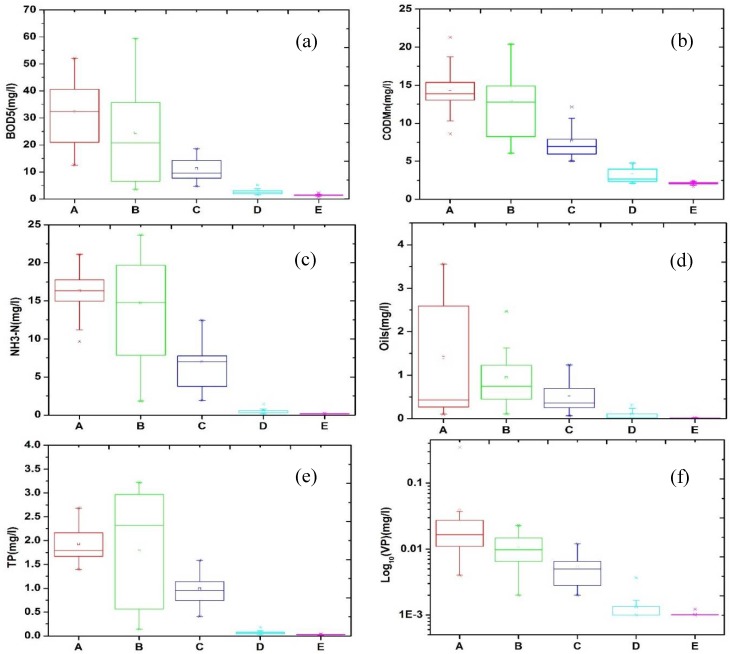
Spatial variations in water quality from 1990 to 2010, (**a**) BOD_5_; (**b**) COD_Mn_; (**c**) NH_3_-N; (**d**) Oils; (**e**) TP; (**f**) VP.

The spatial variations in surface water quality can be clustered into five groups based on sample site during 1990–2010. Cluster A consists of four sites of Shenzhen stream and Buji stream, which can be described as urbanization-polluted stream catchments; water pollution in Cluster A is greater than that in all the other clusters, with 20-year mean values of 14.4 mg/L, 32.30 mg/L, 1.93 mg/L, 0.038 mg/L, 16.32 mg/L, and 1.40 mg/L for COD_Mn_, BOD_5_, TP, VP, NH_3_-N, and Oils, respectively. Cluster B includes seven sites of Xixiang stream, Longgang stream, and Guanlan stream, which are distributed throughout industrial areas of Shenzhen, with 20-year mean values of 12.92 mg/L, 24.54 mg/L, 1.78 mg/L, 0.011 mg/L, 14.78 mg/L, and 0.94 mg/L for COD_Mn_, BOD_5_, TP, VP, NH_3_-N, and Oils, respectively. This cluster can be described as industrial-polluted stream catchments. Cluster C includes three sites of Pingshan stream and Dasha stream, which can be classified as suburban-polluted stream catchments, with 20-year mean values of 7.62 mg/L, 11.21 mg/L, 0.99 mg/L, 0.005 mg/L, 6.98 mg/L, and 0.50 mg/L for COD_Mn_, BOD_5_, TP, VP, NH_3_-N, and Oils, respectively. Cluster D includes three sites of Shenzhen Reservoir, Tiegang Reservoir, and Shiyan Reservoir. These subwatersheds with abundant built-up land can be designated as urbanization reservoir catchments, with 20-year mean values of 3.21 mg/L, 2.76 mg/L, 0.07 mg/L, 0.001 mg/L, 0.54 mg/L, and 0.10 mg/L for COD_Mn_, BOD_5_, TP, VP, NH_3_-N, and Oils, respectively. Cluster E consists of ten small reservoir sites; thus, it comprises natural reservoir catchments. The 20-year mean values of COD_Mn_, BOD_5_, TP, VP, NH_3_-N, and Oils are 2.10 mg/L, 1.46 mg/L, 0.02 mg/L, 0.001 mg/L, 0.17 mg/L, and 0.02 mg/L, respectively (Figure 3). All clusters exhibit significant variations. Notably, a declining trend is shown from Cluster A to E, and Clusters A, B, and C have significantly more pollution than Clusters D and E, suggesting that the pollution of streams is more difficult to control than the pollution of reservoirs. 

Based on water quality parameters, years were clustered into four stages from 1990 to 2010. Stage I included 1991–1994, which was a high point-source pollution stage; Stage II included 1996–1999, which was a point-source pollution control stage; Stage III included 1995, 2000–2001, and 2004–2006 and was a stage of rapid water quality deterioration; Stage IV included 2002–2003 and 2007–2010, which were periods of water quality recovery in Shenzhen. The temporal variations in surface water quality in Shenzhen’s streams indicated that the majority of stream pollution occurred in Stage III.

### 3.2. Landscape Characteristics 

#### 3.2.1. Changes in Watershed Landscape Patterns

**Figure 4:**
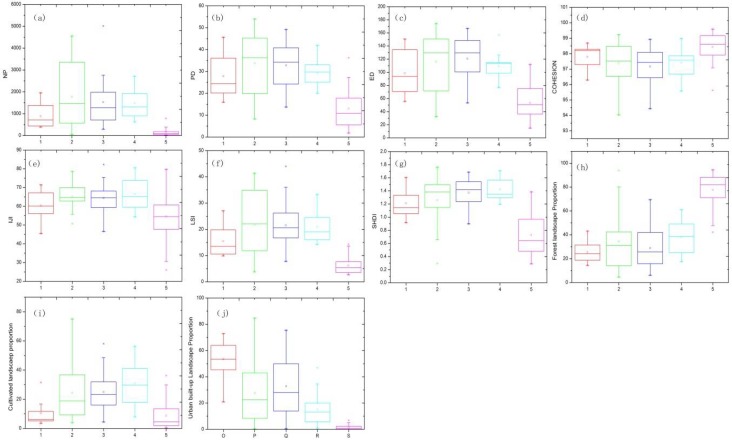
Boxplots of landscape metrics representing the composition and spatial configuration in different spatial clusters from 1990 to 2010, (**a**) NP; (**b**) PD; (**c**) ED; (**d**) Cohesion; (**e**) IJI; (**f**) LSI; (**g**) SHDI; (**h**) ForestP; (**i**) CultiP; (**j**) UrbanP .

Based on the clustering of water quality, the catchments were grouped into five clusters to describe the spatial patterns of the landscape from 1990 to 2010. The variations in the landscape fragmentation, landscape composition, spatial configuration, and patch diversity of Clusters A to E are shown in Figure 4. With accelerated urbanization in Shenzhen, the landscapes in catchments with different water qualities exhibited obvious spatial variations. Overall, the majority of landscape patterns in the catchments of Clusters A to E exhibited inverted U-shaped curves, with exceptions being ForestP, which exhibited an increasing trend, and UrbanP, which exhibited a decreasing slope. The landscapes in Cluster B catchments varied more drastically than those of other catchments, indicating that it underwent rapid urbanization. The boxplots of the landscape pattern indices (Figure 4) include water quality plots (Figure 3) in different clusters, which may suggest that landscape changes have profound effects on water quality degradation in different sub-catchments.

Changes in landscape patterns occurred in different clustered catchments. The variations in the landscape indices of NP, PD, ED, IJI, LSI, SHDI, CultiP and ForestP in Cluster A, where the urban landscape is a basic matrix, were smaller than those in Cluster B, suggesting that a stable stage occurred after the rapid urbanization stage. In Cluster B, the mean values of NP, PD, ED, and LSI were the largest of all five clusters of catchments, suggesting that these watersheds experienced landscape fragmentation, which led to structural complexity as urbanization progressed. However, the variations in IJI, Cohesion, SHDI, ForestP, CultiP, and UrbanP were also much larger than those in the other clusters, indicating that the catchments in Cluster B were severely influenced by human activities. In Cluster C, which included suburban areas, the indices of UrbanP and ForestP were higher than those of Cluster B but lower than those of Cluster A, while CultiP was significantly higher than that of Clusters A and B. In Cluster C, the watershed landscape displayed high fragmentation and diversity, low aggregation, and a highly complex spatial structure, as reflected by NP, PD, ED, and LSI. The landscape pattern changes in Cluster D indicated that it experienced a high degree of landscape urbanization through variations in Cohesion and IJI. Cluster C catchments include water resource protection zones in urban areas with high proportions of agricultural land use and FL and low proportions of urban land. In these areas, water resource protection policies have significantly influenced landscape restoration and the landscape indices of NP, PD, ED, LSI, and SHDI exhibited lower values than those in Clusters B and C. Cluster E is characterized by a natural landscape in an outer suburban area and is a water protection area with the highest aggregation and ForestP values. In this cluster, urban and agricultural landscapes account for low proportions of the total landscape, and the values of NP, PD, ED, LSI, and SHDI were the lowest among all clusters. These findings suggest that the processes of urbanization have had impacts at the sub-catchment landscape, which reflects the gradient pattern of landscape changes from an urban core area to suburban, outer suburban, and, eventually, natural landscape areas.

#### 3.2.2. Temporal Changes in Landscape Patterns in Five Typical Catchments

**Figure 5:**
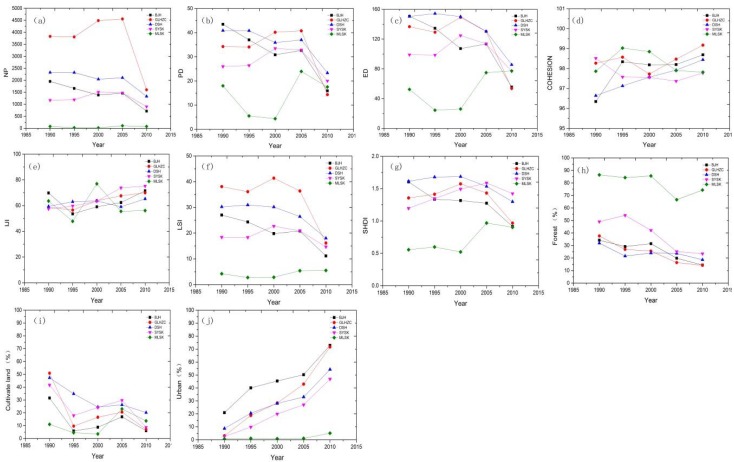
Landscape changes in five typical watersheds from 1990 to 2010, (**a**) NP; (**b**) PD; (**c**) ED; (**d**) Cohesion; (**e**) IJI; (**f**) LSI; (**g**) SHDI; (**h**) ForestP; (**i**) CultiP; (**j**) UrbanP.

To understand the differences in landscape changes according to temporal characteristics, five typical catchments—BJH, GLHZC, DSH, SYSK, and MLSK—were chosen based on water quality indicators at different watershed urbanization levels. All the catchments were located in west-central Shenzhen and exhibited a spatial gradient from urban to suburban areas. Ten landscape configuration and component metrics were used to analyze the characteristics of landscape change in the five typical watersheds (Figure 5). The NP, PD, ED, LSI, SHDI, ForestP and CultiP indices of the five typical watersheds have drastically decreased over the past 20 years, while only Cohesion, IJI and UrbanP increased during the rapid urbanization process. These results suggest that a reduction in landscape fragmentation has occurred in most watersheds and that the transformation of the landscape matrix from a natural landscape to an urban landscape was most significant in the BJH catchment, as reflected by decreasing ED, LSI, and PD values.

The proportion of the urban landscape increased quickly in watersheds that underwent rapid urbanization, such as BJH, followed by increases in industrial and peri-urban catchments such as GLHZC and urban areas with water protection policies, such as SYSK. However, areas of natural water protection exhibited the opposite trend. Landscape pattern changes were significantly different among the typical catchments. The values of NP, ED, LSI, SHDI, and UrbanP in highly urbanized catchments were several times higher than those in natural catchments, such as MLSK, while those of Cohesion and ForestP were several times lower. All these patterns suggest that water quality degradation varies simultaneously at the sub-catchment scale. 

### 3.3. Effects of Landscape Changes on Water Quality Degradation

Table 2 shows the coefficients estimated from the panel data analysis, which indicates a clear relationship between water quality and landscape changes. The R^2^ of the fixed-effects panel model shows that water quality degradation in Shenzhen can largely be determined by the landscape composition and configuration from 1990 to 2010. Specifically, 39–58% of the degradation is reflected by landscape changed during the rapid urbanization process. BOD_5_ was significantly and positively correlated with PD and negatively correlated with ForestP and NP; COD_Mn_ was positively correlated with PD and negatively correlated with NP, ForestP and Cohesion; NH_3_-N was negatively correlated with ForestP and CultiP; and TP was positively correlated with Cohesion and negatively correlated with UrbanP. Moreover, VP exhibited a negative correlation with IJI and CultiP. These five water quality parameters exhibited relationships with changes in the landscape composition, while Oils were more affected by the landscape configuration. These relationships indicate that due to the rapid changes in natural landscapes and BLs, water has become seriously polluted in high-level urbanization catchments and contaminated in water protection areas.

The compositions of various landscape types exhibited different effects on water quality. Notably, forest and cultivated landscapes exhibited negative effects on water quality, while built-up landscapes displayed positive effects based on the various indicators, except for TP. A significant negative correlation was observed for the influences of ForestP and CultiP on COD_Mn_, BOD_5_, NH_3_-N, TP, and VP. The changes in cultivated landscapes contributed to changes of −3.44%, −0.43% and −0.37% in BOD_5_, NH_3_-N and VP, respectively, and the changes in FL accounted for −0.80%, −0.34%, and −0.75% of changes to BOD_5_, COD_Mn_, and NH_3_-N, respectively. With decreases in the areas of natural and semi-natural landscapes, the concentrations of these water quality parameters increase, leading to water quality degradation. Notably, BL exhibited a positive correlation with five water quality parameters, but TP had a significant negative effect on these parameters. The change in the proportion of the urban landscape accounted for a −0.48% change in TP, suggesting that the increase in BL increased the level of contamination. This finding indicates that the influence of decreasing the natural landscape composition is more important than the influence of increasing the urban landscape composition when urban development occurs in an urban watershed.

Changes in the landscape configuration, such as the significant positive correlations observed between PD and three water quality parameters (BOD_5_, COD_Mn_ and Oils), accounted for 4.37%, 2.03% and 5.44% of changes in BOD_5_, COD_Mn_, and Oils, respectively. NP exhibited a significant negative correlation with BOD5, COD_Mn_, and Oils and accounted for changes of −3.44%, −1.63%, and −4.98%, respectively. The impacts of these two landscape fragment indices suggest that landscape fragmentation is an important driver of water quality degradation in urban catchments. IJI had a negative impact on VP (contribution of −1.47%), while LSI only had an impact on Oils (contribution of 6.13%). Cohesion had a small negative influence on COD_Mn_ (−1.87%) and a positive influence on TP (42.97%); SHDI had a negative impact on COD_Mn_ (−1.06%); and ED had no significant impact on any of the six water quality parameters. These results suggest that ED is not an ideal indicator of landscape configuration change as it relates to water quality. However, when landscape fragmentation occurs in urban watersheds, it significantly contributes to changes in water quality.

## 4. Discussion

### 4.1. Relationships between Urban Landscape Changes and Water Quality

Understanding the relationship between landscape patterns and water quality degradation typically requires more than 15 years of continuous water quality data, and it is necessary to distinguish between the impacts of human activities on landscape changes and normal fluctuations in water quality. Our results indicate that the water quality of urban streams is considerably affected by landscape changes. Previous studies focused on the effects of static spatial landscape patterns on water quality in the short term [4,11], ignoring the impacts of landscape changes on water quality [31]. In such studies, the effect of the landscape configuration is often overestimated, and many landscape metrics have been misused by quantifying such relationships using a correlation analysis [32]. However, this research indicates that the effects of landscape composition changes on water quality are generally more important than those of landscape configuration changes [33,34,35].

It is difficult to select landscape metrics as variables to quantify the relationships between landscape characteristics and water quality [36]. Previous studies generally used area/density/edge, shape, isolation, interspersion, connectivity, or diversity metrics to characterize the structure of a watershed or riparian buffer zone [37]. However, the selection of landscape indices could be improved so that changes in watershed landscape patterns and the drivers of urban stream water quality can be reasonably quantified [38]. Our results indicate that landscape composition and configuration metrics and panel regression analysis can improve assessments of water quality degradation.

COD_Mn_ and BOD_5_ were reported as the major pollutants associated with the rapid urbanization process in Shenzhen [39]. Previous studies in Shenzhen have demonstrated that the urban landscape composition is nonlinearly related to water quality parameters [18]. Therefore, linear regression methods, such as stepwise regression or correlation analysis, are unsuitable for quantifying these relationships, although they are currently the most popular methods used to analyze the relationships between water quality and landscape patterns [40]. To quantify these nonlinear relationships and avoid collinearity, panel regression analysis based on the log transformation of variables was employed in this study, indicating that this method can effectively quantify these relationships.

Other studies have simulated the pollution load based on the landscape or land use, such as studies based on SWAT, HSPF, or L-THIA, with the purpose of quantifying and reducing water quality pollution [41]. However, the pollutant load does need to be directly discharged into streams to create water pollution issues due to landscape changes. Environmental engineering measures can only slow water quality deterioration and cannot completely restore river water quality to its natural state [42]. Thus, engineering measures can quickly reduce pollutant levels but cannot eliminate the cumulative effects of landscape changes. If stream water quality is to be restored, it is necessary to provide a scientific basis for establishing environmental policy and to mitigate the negative effects of landscape changes on water quality.

### 4.2. Policy Implications for Water Quality Management 

Understanding the relationship between regional water quality and landscape change is important for providing evidence-based policy recommendations for the sustainable governance of water quality resources in both developing and developed urban areas [43]. Heterogeneous drivers of water quality reflect the necessity for diverse resource policies that account for differences in water quality in urban stream systems and promote the recovery or restoration of water quality. Model evaluations demonstrate that many parameters, such as COD_Mn_ and BOD_5_, are highly correlated with PD and the natural landscape composition. Therefore, local management plans should focus on increasing the extent and density of natural landscape changes and on controlling the expansion of built-up landscapes to potentially improve water quality in urban stream systems. Landscape restoration should be planned to appropriately increase the proportion of the natural landscape, which can efficiently reduce the risk of non-point-source pollution to water bodies. Therefore, at the sub-watershed scale, landscape pattern optimization is one management method that should be considered. 

## 5. Conclusions

In this study, the relationships between water quality and landscape changes were investigated during the rapid urbanization of Shenzhen using panel regression analysis. The results show that:

(1) A clear spatial and temporal distribution of water quality along with urban landscape change was identified in Shenzhen, which considerably affected stream water quality degradation during urbanization.
(2) Water quality is sensitive to different landscape changes, particularly changes in urban and forest areas. Landscape composition change is an important process that affects water quality degradation; specifically, decreases in the proportions of cultivated and forest landscapes influence water quality changes more than increases in the proportions of urban built-up landscapes.
(3) The landscape changes explained 39–58% of the variations in water quality based on different water quality parameters. The landscape configuration also influences water quality degradation, but landscape metrics are not highly correlated with water quality parameters, except for landscape fragment indices, such as NP and PD, which play significant roles.
(4) Water quality degradation and landscape changes have kept pace with each other in sub-catchment, so reducing landscape fragmentation and enhancing the natural landscape composition at the watershed scale are of vital importance for improving water quality. Therefore, optimizing the landscape pattern is an alternative strategy for alleviating urban water quality degradation. The results of this study reflect the relationships between urban landscape development and stream water quality and can be used to improve water quality in urban areas.

## Figures and Tables

**Table 1:** Water quality characteristics in Shenzhen from 1990 to 2010.

Water Quality Indicator	Obs.	Mean	S.D.	CV	Max.	Min.	National Quality Standards for Surface Waters in China (GB3838-2002)				
							Level I	Level II	Level III	Level IV	Level V
COD_Mn_	540	6.21	6.02	0.97	27.90	1.09	≤2	4	6	10	15
BOD_5_	540	10.33	16.35	1.58	100.1	0.50	<3	3	4	6	10
NH_3_-N	540	5.44	8.02	1.47	37.56	0.03	≤1.5	0.5	1.0	1.5	2.0
VP	540	0.01	0.04	4.86	0.67	0.00	<0.002	0.002	0.005	0.01	0.1
Oils	540	0.40	0.76	1.89	5.14	0.01	<0.05	0.05	0.05	0.5	1.0
TP	540	0.67	1.04	1.55	4.95	0.01	≤0.02	0.1	0.2	0.3	0.4

**Table 2:** Relationships between water quality and landscape changes via fixed-effects panel regression analysis using log-log variables.

Landscape Indicator	Water Quality Indicator											
	Log(BOD_5_)		Log(COD_Mn_)		Log(NH_3_-N)		Log(TP)		Log(VP)		Log(Oils)	
	Coefficient	Std. Err.	Coefficient	Std. Err.	Coefficient	Std. Err.	Coefficient	Std. Err.	Coefficient	Std. Err.	Coefficient	Std. Err.
Log(NP)	−3.44 **	1.10	−1.63 **	0.60	−0.78	1.23	0.58	1.77	−0.95	1.17	−4.98 **	1.62
Log(PD)	4.37 ***	1.22	2.03 **	0.67	1.26	1.37	−0.93	2.03	1.97	1.30	5.44 **	1.78
Log(ED)	−2.58	1.82	−1.23	0.99	1.76	2.04	5.27	3.29	−0.45	1.94	−2.57	2.67
Log(LSI)	3.11	1.89	1.74	1.04	−1.69	2.12	−4.90	3.17	−0.50	2.02	6.13 *	2.83
Log(IJI)	−0.51	0.71	−0.57	0.39	−0.11	0.80	1.82	1.18	−1.47 *	0.76	0.61	1.04
Log(Cohesion)	4.32	11.77	−1.87 *	6.46	12.65	13.20	42.97 *	18.51	16.07	12.56	15.53	17.25
Log(SHDI)	−1.05	0.90	−1.06 *	0.49	−0.68	1.01	−0.36	1.44	0.99	0.96	−1.87	1.32
Log(CultiP(%))	−0.21	0.17	−0.009	0.093	−0.43 *	0.19	−4.82	0.26	−0.37 *	0.18	−3.83	0.26
Log(ForestP(%))	−0.80 **	0.31	−0.34 *	0.17	−0.75 *	0.34	−3.63	0.46	−0.03	0.33	0.23	0.45
Log(UrbanP(%))	0.14	0.11	0.07	0.06	0.01	0.13	−0.48 **	0.18	0.13	0.12	0.16	0.187
Constant	−0.94	54.68	18.87	30.00	−55.35	61.37	−215.47	86.84	−69.32	58.37	−65.38	80.24
σ_u_	0.482		0.395		1.011		1.142		0.533		0.527	
σ_e_	0.733		0.403		0.823		1.099		0.783		1.067	
*p*	0.301		0.491		0.601		0.519		0.317		0.195	
Within R^2^	0.16		0.10		0.14		0.05		0.14		0.16	
Between R^2^	0.77		0.74		0.69		0.55		0.74		0.83	
Overall R^2^	0.58		0.58		0.56		0.39		0.50		0.57	

*** *p* < 0.001; ** *p* < 0.01; * *p* < 0.05.
